# Dual-positive gastric cancer: an extremely malignant subtype of gastric cancer with high serum alpha-fetoprotein and carcinoembryonic antigen concentrations

**DOI:** 10.3389/fonc.2024.1514069

**Published:** 2025-01-20

**Authors:** Weixun Xie, Chengyu Hu, Hongming Liu, Zelai Wu, Bixian Luo, Xiaoying Wu, Chuanlei Tuo, Ziyin Deng, Han Liang, Yong Liu, Weihua Gong

**Affiliations:** ^1^ Department of Surgery, Second Affiliated Hospital of School of Medicine, Zhejiang University, Hangzhou, China; ^2^ Department of General Surgery, Tongde Hospital of Zhejiang Province, Hangzhou, China; ^3^ Department of Gastric Surgery, Tianjin Medical University Cancer Institute & Hospital, National Clinical Research Center for Cancer, Key Laboratory of Cancer Prevention and Therapy, Tianjin Key Laboratory of Digestive Cancer, Tianjin, China; ^4^ Liangzhu Laboratory, Zhejiang University Medical Center, Hangzhou, Zhejiang, China

**Keywords:** gastric cancer, AFPGC, AFP, CEA, chemotherapy

## Abstract

**Background:**

Alpha-fetoprotein-producing gastric cancer (AFPGC) is a highly malignant subtype of gastric cancer, but solely alpha-fetoprotein may fail to accurately predict the prognosis. Although the utilization of multi tumor markers could improve stratified patient management, such research in AFPGC is still blank. This study seeks to evaluate whether combining multiple tumor markers can enhance risk stratification and identify AFPGC subtypes with poor prognosis.

**Methods:**

We first screened for patients with elevated serum CEA levels within the AFPGC cohort and evaluated their prognosis. Tumor characteristics and overall health conditions were analyzed to identify factors contributing to CEA elevation. Finally, the treatment responses of this group to different treatment modalities were also reviewed.

**Results:**

Approximately 45% of gastric cancer patients with elevated serum AFP also show increased CEA levels, classifying them as the dual-positive gastric cancer (DPGC) subgroup. These patients exhibit significantly shorter overall survival, heightened metastasis risk, and are more susceptible to systemic inflammation, immune response dysregulation, malnutrition, and cancer-related thrombosis. The elevation in serum CEA levels may indicate gastric cancer liver metastasis and increased neutrophils. While surgery is optimal for AFPGC, DPGC patients benefit significantly from immunotherapy combined with chemotherapy.

**Conclusions:**

In AFPGC, combining serum AFP and CEA offers a more accurate prognosis. The poor prognosis in DPGC may be associated with aggressive local properties and systemic complications. Liver metastases and increased neutrophils are associated with increased serum CEA in AFPGC. Immunochemotherapy is a viable option for DPGC patients who cannot undergo surgery.

## Introduction

Serum proteins, which holistically reflect both systemic and tumor-specific characteristics, are easy to obtain and detect, so it is possible to establish a serum protein classification for gastric cancer (1). Secretory proteins released by tumor cells are potentially a major source of serological tumor markers because secreted protein proteins have the greatest potential to enter the circulation (2). One such secretory protein, the alpha-fetoprotein (AFP), has been employed in the diagnosis of various cancers, including hepatocellular carcinoma, yolk sac tumor, tumors of gonadal origin, and specific types of gastric cancers (GC) (3).

Alpha-fetoprotein producing gastric carcinoma (AFPGC) is an uncommon subtype of gastric cancer that exhibits elevated levels of AFP, constituting 1.3–5.4% of gastric cancer cases (4). The 5-year survival rate for all stages of AFPGC ranged from 8.3% to 11.9%, which was even lower than the 5-year survival rate for advanced gastric cancer (5). AFPGC is notorious for its poor prognosis and aggressive clinical features such as a high tendency for vascular invasion, liver metastasis, and lymph node involvement (6). Although studies have indicated that AFP contributes to the aggressive behavior of AFPGC, whether it could serve as a prognostic marker for patient survival is controversial (7–10).

In our previous study, we found that the aggressive biological characteristics of AFPGC were closely related to abnormalities in other tumor biomarkers. Therefore, we proposed that the combined use of multiple biomarkers was of great significance for a comprehensive assessment of AFPGC patients (11). Carcinoembryonic antigen (CEA) is routinely measured as a serum marker in gastric cancer and has been identified as an independent predictor of poor outcomes in the early stage (12). Li Y. et al. reported that the serum CEA value of AFPGC was significantly higher than that of the other gastric cancer, while other gastrointestinal tumor markers, such as CA19-9, CA724, and CA125 showed no difference (13). Li N. et al. reported that AFPGC patients with normal CEA levels experienced longer median overall survival, suggesting that CEA could be a valuable prognostic indicator for this cancer subtype (5). Nevertheless, there is a dearth of clinical studies focusing on AFPGC patients with concurrent elevations in serum CEA.

In our research, we assessed 127 patients with AFPGC treated at our institution, collecting their clinicopathological information and prognostic data. We named AFPGC with high CEA expression as dual-positive gastric cancer (DPGC). Our analysis aimed to illuminate the prognosis, peculiarities and treatments of DPGC. To the best of our knowledge, this may be the largest clinical study for AFPGC to date.

## Methods

### Patients

A total of 127 patients with pre-treatment elevated serum AFP at National Clinical Research Center for Cancer (Tianjin, China) between August 2017 and May 2023 were included in this study. Of these, thirty-five patients presented with elevated serum CEA levels exceeding 5 ng/mL.

The inclusion criteria were as follows:

Pathological confirmation of gastric adenocarcinoma;
Elevation of serum AFP (>7 ng/mL) prior to any antitumor treatment;
Absence of concurrent conditions known to elevate serum AFP, such as primary hepatocellular carcinoma, germ cell tumor, chronic hepatitis, or cirrhosis.

The exclusion criteria included:

A history of other cancers;
Previous treatment with chemoradiation, chemotherapy, interventional therapy, or any antitumor therapy before hospital admission;
Lack of a pre-treatment serum CEA test.

### Data collection and definition

Upon admission, all patients underwent blood tests within one week prior to initiating treatment. The threshold values for serum AFP, CEA, carbohydrate antigen 125 (CA125), carbohydrate antigen 19-9 (CA19-9), carbohydrate antigen 72-4 (CA72-4), carbohydrate antigen 242 (CA242), and lactate dehydrogenase (LDH) were set at 7 ng/mL, 5 ng/mL, 35 U/mL, 6.9 U/mL, 20 IU/mL, and 250 U/L, respectively, as per the manufacturer’s recommendations. The eighth edition of the Tumor-Node-Metastasis (TNM) staging system developed by American Joint Committee on Cancer was used to stage tumors in all patients (14).

### Kaplan- Meier survival curves

A comprehensive follow-up was conducted on a cohort of 127 cases, albeit with the unfortunate loss of some to follow-up. Out of the 109 patients successfully tracked, 29 underwent radical surgical procedures, 34 were treated with chemotherapy, 29 received a combination of immunotherapy and chemotherapy, 14 initiated their treatment with preoperative neoadjuvant chemotherapy, and 3 patients opted for self-discharge. Kaplan- Meier Survival Curves was plotted by https://www.bioinformatics.com.cn, an online platform for data analysis and visualization.

### Statistical analysis

Chi-squared tests, and Student’s t-test tests were applied to the clinical data, with a two-tailed p-value of < 0.05 indicating statistical significance (*p < 0.05, **p < 0.01, ***p < 0.001). All statistical computations were performed with SPSS software, version 27.0 (SPSS Inc., Illinois, USA).

## Results

### AFPGC patients with elevated serum CEA are associated with a poorer prognosis

In our cohort of 127 AFPGC patients, the majority were male (97 individuals) with a median age of 60.4 years, ranging from 27 to 90. The tumors were predominantly located in the distal third of the stomach (69 patients), followed by the upper third (34 patients), and the middle third (19 patients). Quite notably, 5 patients presented with extensive disease affecting more than two-thirds of the stomach. Concerning the surgical interventions applied, 37 patients underwent curative surgery, while only 3 received palliative operations, and 6 patients opted for self-discharge. Chemotherapy was administered to 69 patients, which included 14 who received neoadjuvant chemotherapy prior to undergoing curative surgery and 32 who were treated with a combination of immunotherapy and chemotherapy (**Table 1**).

**Table 1:** General characteristics of the patient cohort.

	DPGC	SPGC	*P-value*
Gender, n (%)			0.420
Male	46 (80.7%)	51 (72.9%)	
Female	11 (19.3%)	19 (27.1%)	
Total	57	70	
Age, n (%)			0.174
≥60	39 (68.4%)	41 (58.6%)	
<60	18 (31.6%)	29 (41.4%)	
Total	57	70	
Blood Type, n (%)			0.948
A	16 (30.2%)	23 (33.3%)	
B	18 (34.0%)	20 (28.9%)	
AB	6 (11.3%)	8 (11.6%)	
O	13 (24.5%)	18 (26.1%)	
Total	53	69	
Treatment, n (%)			0.043
Radical surgery	8 (14.0%)	29 (41.4%)	
Non-surgical treatment	39 (68.4%)	30 (42.9%)	
Others	4 (7.0%)	5 (7.1%)	
Total	57	70	
Location, n (%)			0.197
Upper third	18 (31.6%)	16 (22.9%)	
Middle third	6 (10.5%)	13 (18.6%)	
Distal third	30 (52.6%)	39 (55.7%)	
Two thirds or more	3 (5.3%)	2 (2.9%)	
Total	57	70	
Differentiation, n (%)			
Poor	31 (54.4%)	56 (80.0%)	0.019
Moderate	11 (19.3%)	6 (8.6%)	
Total	42	62	
Her2 expression, n (%)			0.011
Yes	19 (52.8%)	32 (65.3%)	
No	29 (47.2%)	17 (34.7%)	
Total	48	49	

χ², Chi-square test; DPGC, dual-positive gastric cancer; SPGC, single positive gastric cancer.

**Figure 1:**
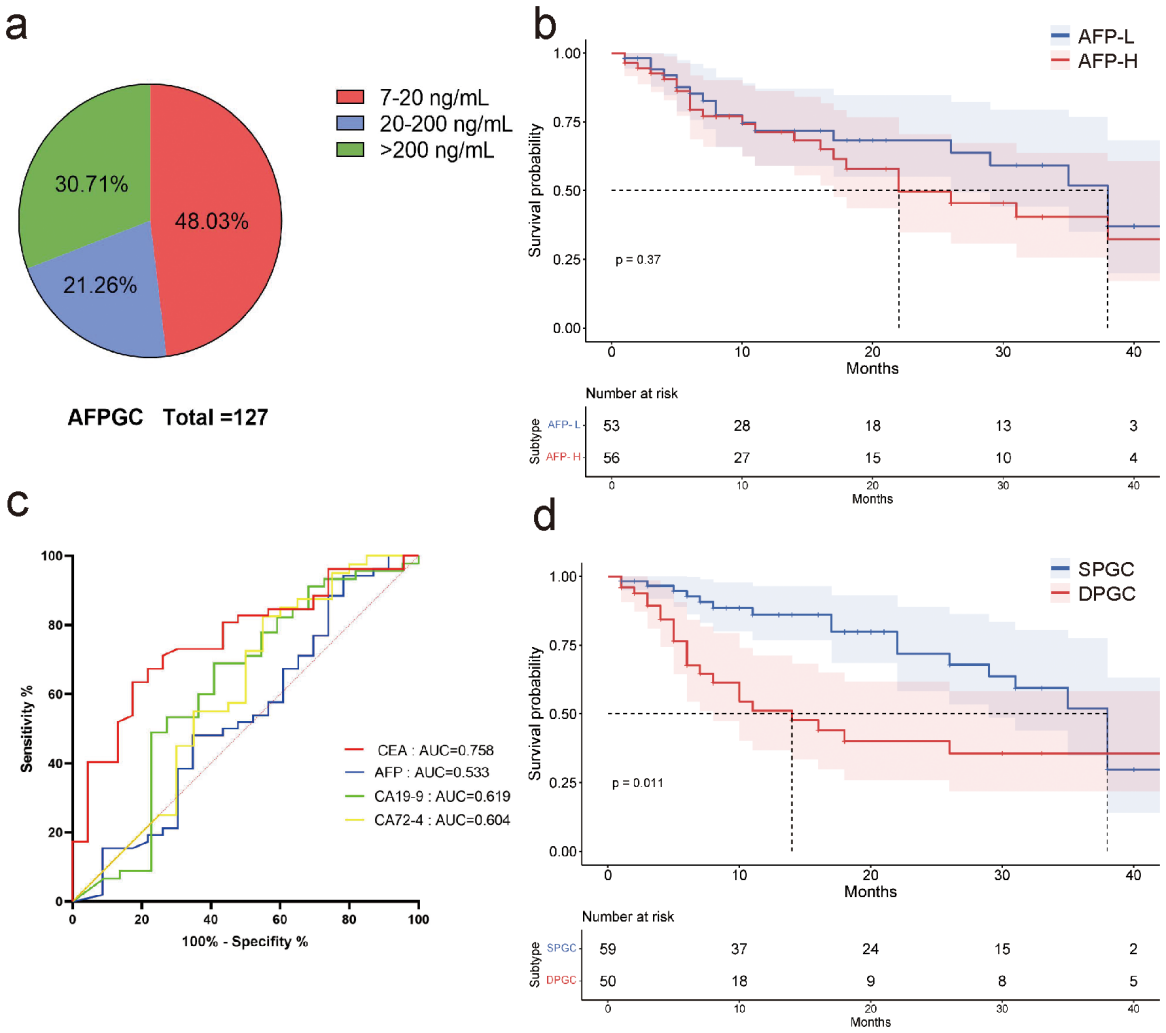
AFPGC patients with elevated serum CEA are associated with a poorer prognosis. **(A)** nearly half of the patients with elevated serum AFP had serum AFP concentrations between 7 and 20 ng/mL; **(B)** high levels of serum AFP did not affect patient survival; **(C)** in AFPGC, serum CEA more accurately predicts the prognosis of patients.; **(D)** survival of DPGC patients was significantly lower than that of SPGC patients. AFP, alpha-fetoprotein protein; CEA, carcinoembryonic antigen; AFPGC, alpha-fetoprotein producing gastric cancer; SPGC, single-positive gastric cancer; DPGC, dual-positive gastric cancer.

In our study, nearly half of the patients had mildly elevated pretreatment serum AFP (7-20ng/mL) (**Figure 1A**). We found elevated serum AFP levels (AFP≥20 ng/mL, AFP-H) did not affect patient survival (P=0.37, **Figure 1B**). Nevertheless, serum CEA demonstrated superior predictive ability for survival compared to other serum gastrointestinal tumor markers, including AFP, CA19-9, as well as CA72-4, with AUC values of 0.758, 0.533, 0.619 and 0.604, respectively (**Figure 1C**).

Consequently, we postulate that serum CEA promotes tumor progression in AFPGC and patients with elevated serum CEA may have a distinct prognosis. We have categorized this cohort as ‘dual positive gastric cancer (DPGC)’, while those without elevated CEA are termed ‘single positive gastric cancer (SPGC)’. Notably, the survival rate for DPGC patients was substantially lower than for those with SPGC, suggesting a grimmer outlook for individuals with this particular subtype of gastric cancer (P=0.011, **Figure 1D**).

### DPGC is a remarkably aggressive of gastric cancer

**Figure 2:**
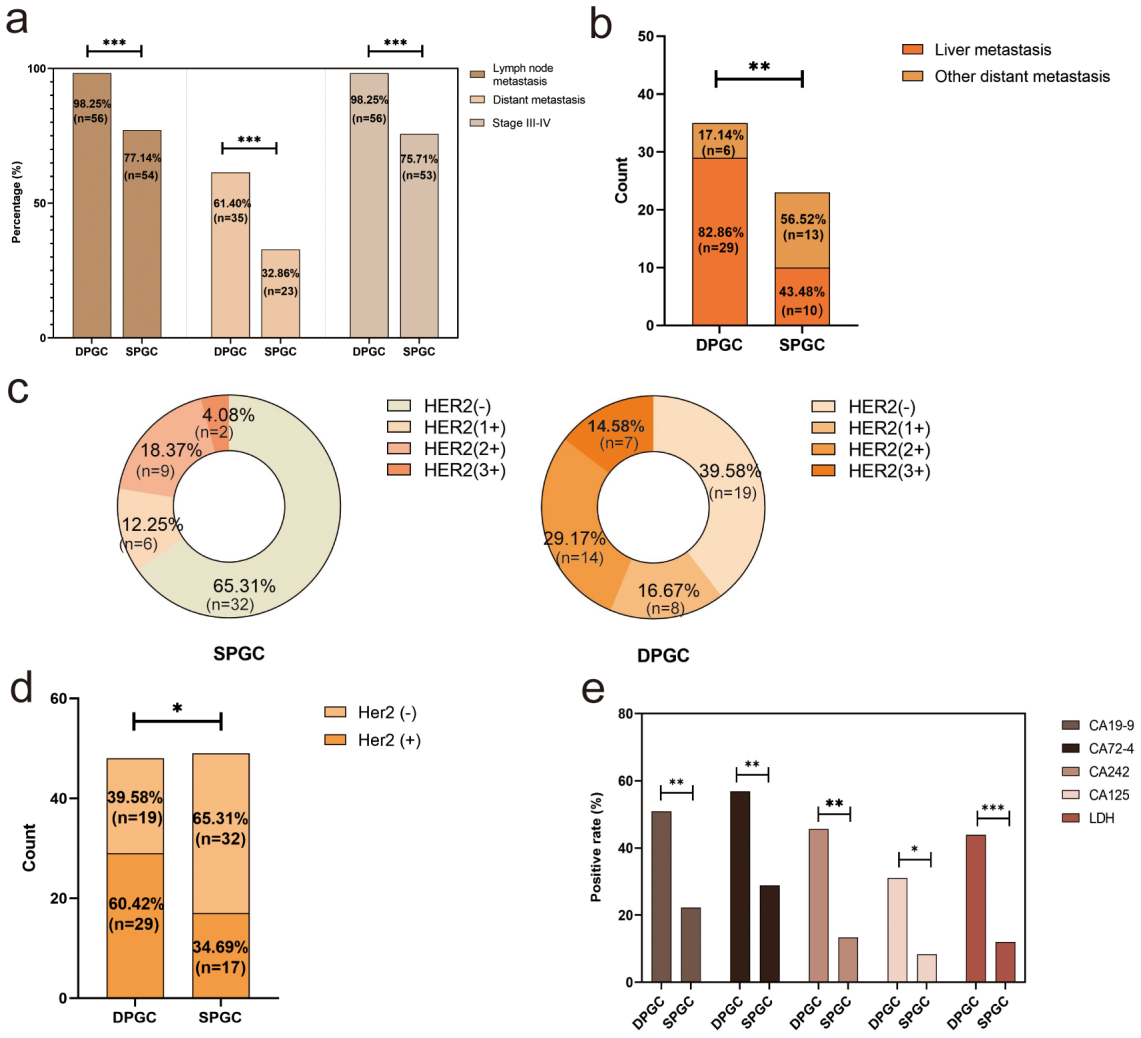
DPGC is a remarkably aggressive of gastric cancer. **(A)** DPGC patients had a significantly higher proportion of high-grade clinical stage, distant metastasis, liver metastasis; **(B)** DPGC exhibits organotropism for liver metastases; **(C)** Expression of HER2 in SPGC and DPGC; **(D)** Significantly more DPGC patients than SPGC patients expressed HER2; **(E)** DPGC patients had significantly higher positivity for serum CA19-9, CA72-4, CA242, CA125, and LDH. HER2, human epidermal growth factor receptor 2; LDH, lactate dehydrogenase. The asterisks indicate significance: * P < 0.05, ** P < 0.01, *** P < 0.001.

Our further investigative work into the malignancy of DPGC tumors found that the majority (98.25%) were classified as TNM stage III-IV, compared to the lesser proportion (75.71%) seen in SPGC cases (P<0.001, **Figure 2A**). This trend was also evident in the rates of lymph node metastasis and distant metastasis: 98.25% and 61.40% in DPGC patients, respectively, substantially exceeding the 77.14% and 32.86% observed in the SPGC group (P<0.001 for both, **Figure 2A**). Furthermore, a possible organ preference for metastasis in DPGC was hint, as indicated by 82.86% DPGC patients with metastasis presenting with liver involvement, in sharp contrast to the 43.48% rate among SPGC counterparts (P=0.004, **Figure 2B**). Additionally, a greater number of DPGC patients exhibited HER2 expression, which is associated with more aggressive tumor behavior (60.42% vs. 34.69%, P=0.011, **Figures 2C, D** **) (** 15).

Classical tumor markers such as CA19-9, CA72-4, CA242, and CA125 play significant roles in the diagnosis and monitoring of gastric cancer (16, 17). When comparing DPGC patients to those with SPGC, as shown in **Figure 2E**, there was a marked increase in the proportion of individuals with elevated levels of these markers: CA19-9 (50.94% vs. 22.22%, P=0.002), CA125 (31.03% vs. 8.33%, P=0.026), CA72-4 (56.86% vs. 28.85%, P=0.005), and CA242 (45.65% vs. 13.33%, P=0.001). Moreover, lactate dehydrogenase (LDH), a tumor biomarker linked to cancer metabolism, invasion, and immune system evasion, showed a higher prevalence of increased levels in the DPGC group (43.9%) compared to the SPGC group (12%, P<0.001).

### DPGC is pertinent to elevated systemic immune-inflammation response, malnutrition and thrombosis

**Figure 3:**
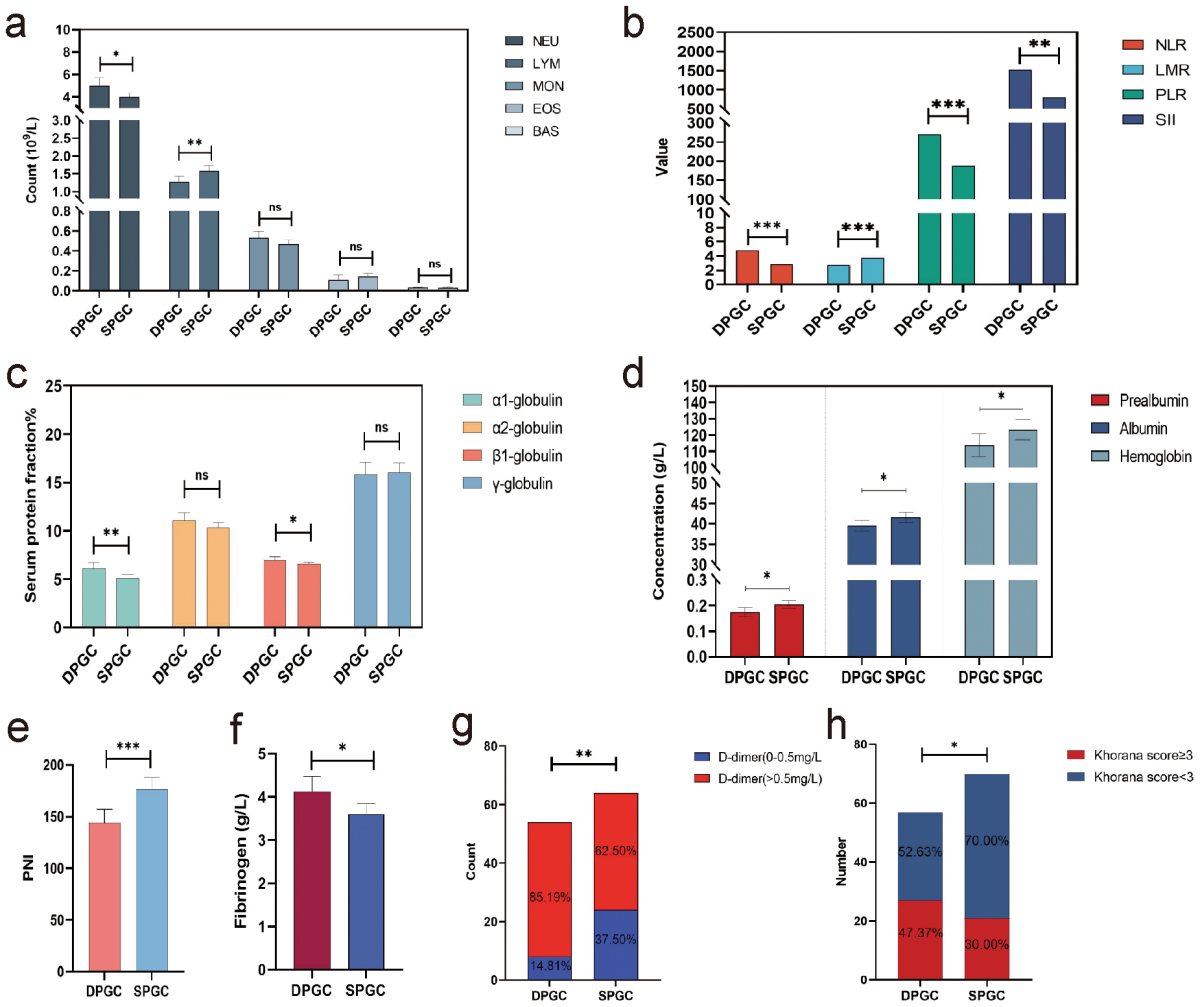
DPGC is pertinent to elevated systemic immune-inflammation response, malnutrition and thrombosis. **(A)** the percentage of neutrophils in the blood of patients with DPGC was significantly increased, while the percentage of lymphocytes and eosinophils was significantly decreased; **(B)** In DPGC, the NLT, PLR, and SII values were significantly higher, while LMR was significantly lower; **(C)** The percentage of α1-globulin and β1-globulin in the plasma of patients with DPGC was high compared with that of patients with SPGC; **(D)** Patients with PGC have significantly reduced levels of serum prealbumin, albumin, and hemoglobin; **(E)** Compared to SPGX patients, DPGC patients had significantly lower PNI values; **(F)** The concentration of fibrinogen was significantly higher in patients with DPGC; **(G)** The concentration of D-dimer was significantly higher in patients with DPGC; **(H)** The proportion of people with a Khorana score ≥3 was significantly higher in DPGC than in SPGC. NEU, neutrophil; LYM, lymphocyte; MON, monocyte; EOS, eosinophil; BAS, basophil; NLR, neutrophil-to-lymphocyte ratio; LMR, lymphocyte-to-monocyte ratio; PLR, plate-to-lymphocyte ratio; SII, systemic inflammation immune index; PNI, prognosis nutritional index. The asterisks indicate significance: * P < 0.05, ** P < 0.01, *** P < 0.001, ns P > 0.05.

Cancer-related inflammation is acknowledged as a critical intermediary in tumorigenesis, frequently preceding the onset of cancer and promoting its advancement (18). Notably, various hematological parameters are considerably altered in DPGC patients, including reduced lymphocyte count and increased neutrophil count (mean 1.27×10^9^/L vs. 1.58×10^9^/L, P=0.003; mean 4.97×10^9^/L vs. 3.94×10^9^/L, P=0.013, **Figure 3A**). Neutrophil-to-lymphocyte ratio (NLR), lymphocyte-to-monocyte ratio (LMR) and platelet-to-lymphocyte ratio (PLR) are cancer-related inflammatory markers that could predict prognoses after treatment in cancer patients (19, 20). We observed that the NLR, PLR, and LMR underwent considerable changes in DPGC patients (mean 4.80 vs. 2.85, P<0.001; mean 269.43 vs. 187.63, P=0.001; mean 2.71 vs. 3.77, P<0.001, **Figure 3B**). The systemic immune-inflammation index (SII), derived from lymphocyte, neutrophil, and platelet counts, was notably higher in DPGC (mean 1525.32 vs. 794.32, P=0.005, **Figure 3B**), suggesting an association with poorer survival outcomes in gastric cancer (21, 22).

In DPGC patients, positive acute phase reactant proteins (APPs), including α1-globulin and β1-globulin, are significantly elevated (mean values of 6.11% vs. 5.07%, P=0.005; and 6.98% vs. 6.58%, P=0.030, respectively), as shown in **Figure 3C**. The notable decreases of negative APPs, such as albumin and prealbumin in DPGC (mean 0.17g/L vs. 0.20g/L, P=0.013; 39.64g/L vs. 41.62g/L, P=0.030, **Figure 3D**) suggest more pronounced APR activation.

Studies have shown that there is a strong relationship between immune dysfunction and malnutrition, and that the two are mutually reinforcing (23). In our cohort, other than serum albumin and prealbumin, DPGC patients presented reduced hemoglobin levels compared to SPGC patients (mean 113.74g/L vs. 123.37g/L, P=0.042, **Figure 3D**). The prognostic nutritional index (PNI), recognized as a predictive marker for survival in gastric cancer, was also significantly lower in the DPGC group (mean 143.67 vs. 176.59, P<0.001, **Figure 3E**) (24). Collectively, these nutritional indicators highlight the compromised nutritional status of DPGC patients, which may contribute to their unfavorable prognosis.

Cancer-associated thrombosis (CAT) ranks as a leading cause of mortality in cancer patients, surpassed only by cancer progression itself (25). Both fibrinogen and D-dimer levels in DPGC surpassed the upper normal limit and were significantly elevated compared to SPGC (mean 4.13g/L vs. 3.61g/L, P=0.016; and 2232.02 ng/mL vs. 1323.53 ng/mL, P=0.016, respectively, **Figures 3F, G**).The Khorana score, a reliable measure for ascertaining venous thromboembolism risk in cancer patients, indicated that a greater proportion of DPGC patients were classified as high-risk (Khorana score ≥3) in comparison to SPGC patients (47.37% vs. 30.00%, P=0.045, **Figure 3H**) (26).

### Factors impacting CEA level in AFPGC

**Figure 4:**
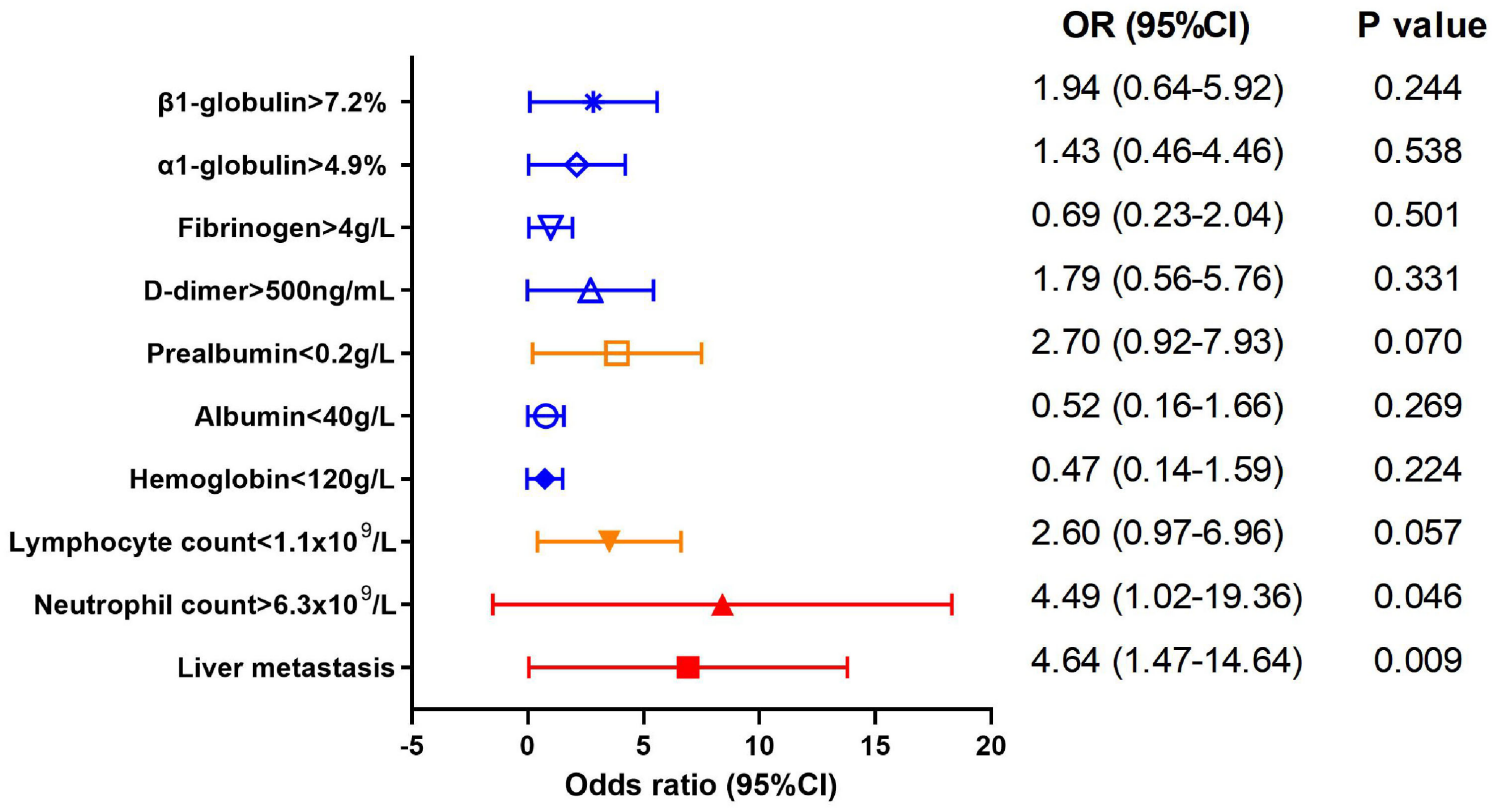
Factors impacting CEA level in AFPGC. Liver metastasis and increased neutrophil were positively correlated with elevated CEA level.

Thereafter we explored the factors contributing to the elevated CEA level (>5 ng/mL) in AFPGC using a binary regression model, as depicted in **Figure 4**. The results showed that liver metastasis and increased neutrophil were positively correlated with elevated CEA level (OR 4.64, 95%CI (1.47-14.64), P=0.009; OR 4.49, 95%CI (1.02-19.36), P=0.046). Additionally, AFPGC patients with lymphopenia may also exhibit DPGC (OR 2.60, 95%CI (0.97-6.96), P=0.057).

### DPGC patients benefit from immunotherapy combined with chemotherapy

**Figure 5:**
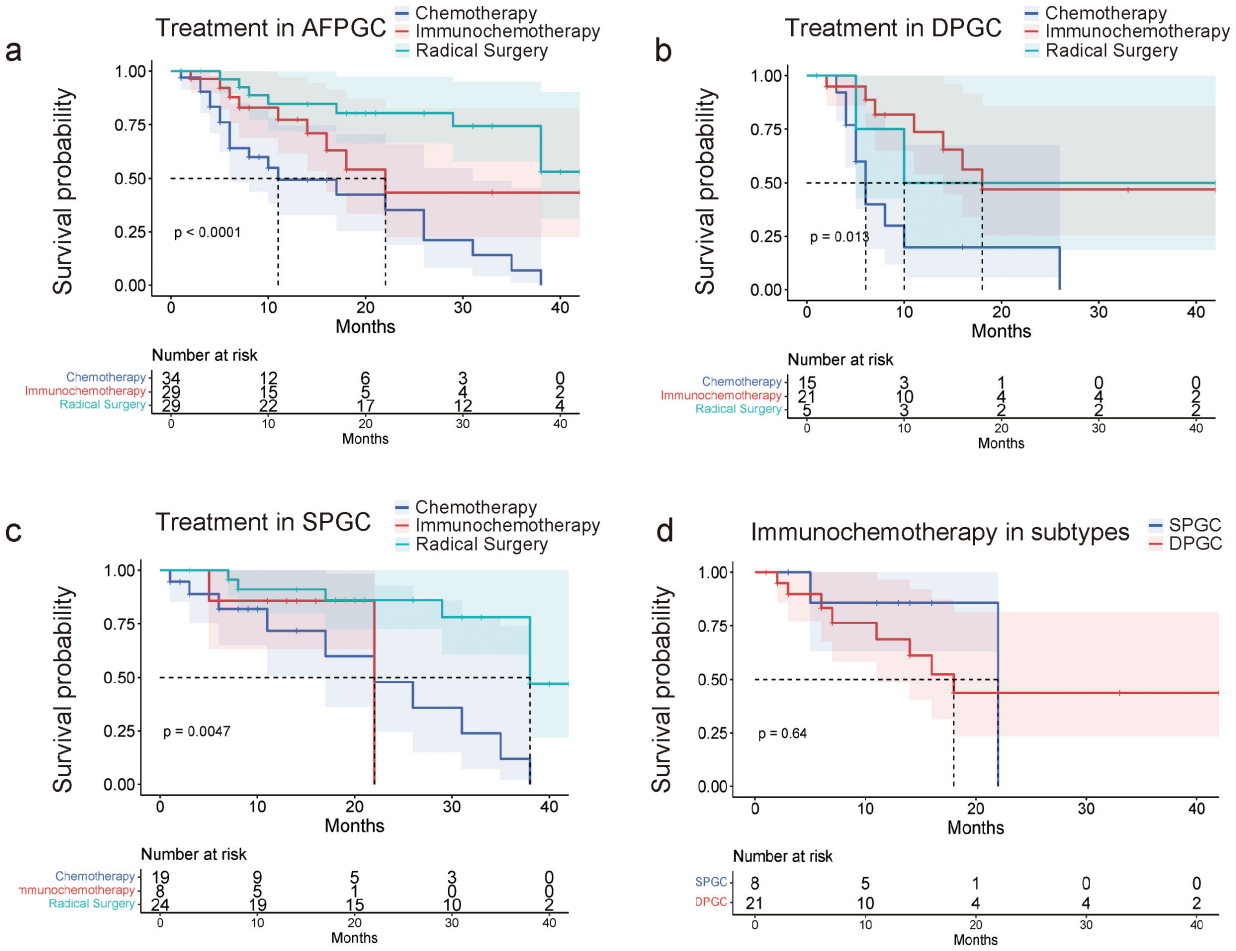
DPGC patients benefit from immunotherapy combined with chemotherapy. **(A)** AFPGC patients do best with surgery, and immunochemotherapy is superior to chemotherapy; **(B)** DPGC patients could significantly benefit from immunochemotherapy; **(C)** Immunochemotherapy does not prolong OS of SPGC patients; **(D)** DPGC patients had significantly longer OS after receiving immunochemotherapy compared to SPGC patients.

We continue to explore whether there are differences in the response to treatment regimens between two subtypes of gastric cancer. Initially, radical surgery remains the optimal treatment option for all AFPGC patients. Among non-surgical treatments, patients who received combined immunotherapy and chemotherapy have a longer overall survival (OS) compared to those who only received chemotherapy (median OS 22.0 months vs. 11.0 months, P=0.024, **Figure 5A**). Specifically, for the DPGC and SPGC subtypes of gastric cancer, while radical surgery continues to be the best treatment option for both, the response to the combined immunotherapy and chemotherapy regimen is significantly different. Patients with DPGC benefit more substantially from combined immunotherapy and chemotherapy than chemotherapy, while those with SPGC do not (median OS 18.0 months vs. 6.0 months, P=0.005, median OS 22.0 months vs. 22.0 months, P=0.91, **Figures 5B, C**). There is no significant difference in overall survival between DPGC and SPGC patients who received combined immunotherapy and chemotherapy (median OS 18.0 months vs. 22.0 months, P=0.64, **Figure 5D**).

## Discussion

AFPGC is designated by the World Health Organization’s (WHO) classification of digestive system tumors as an infrequent form of GC with elevated AFP expression (27). However, whether high serum alpha-fetoprotein is an independent prognostic indicator in AFPGC remains controversial (9, 28, 29). CEA belongs to a family of molecules implicated in the progression and metastasis of cancer (30). Furthermore, a study conducted by He et al. indicated that CEA elevation might be a potential independent predictive factor for prognosis of AFPGC (5). Therefore, gastric cancer with both elevated levels of AFP and CEA may have a worse prognosis.

In this study, AFPGC is characterized by serum AFP exceeding the normal upper limit, obviating the need for AFP immunohistochemistry in tumor tissues. This definition provides a more precise and practical criterion for clinical application. Our findings reveal that, in AFPGC cases, serum CEA (>5 ng/mL) rather than serum AFP (>20ng/mL) is a superior predictor of one-year patient survival. Such an observation challenges the traditional threshold for defining AFPGC at serum AFP levels above 20 ng/mL—a criterion that may unnecessarily exclude patients with slightly elevated serum AFP levels who still exhibit a poorer prognosis. By employing simultaneous testing of serum CEA and AFP, we have ascertained that patient exhibiting elevations of both biomarkers exhibit poorer prognoses compared to those with an isolated rise in serum AFP. This has led us to propose a new molecular subtyping for gastric cancer, predicated on serum AFP and CEA levels, designating cases with elevated levels of both as DPGC.

Next, for patients with DPGC, we conducted an analysis on both the local tumor characteristics and the overall systemic condition of the patients. Patients with DPGC are more susceptible to tumor metastasis, including lymph node and distant metastases. This may be attributed to the fact that CEA has already been identified as a metastatic driver. Overexpression of CEA enriches the expression patterns of epithelial genes, thereby facilitating the growth of tumors at metastatic sites (31). Meanwhile, DPGC exhibits a distinct organotropism towards the liver during metastasis. We speculate that this may be due to the tumor’s increased expression of CEA in these patients, given that CEA can directly bind to CEA receptors on the surface of hepatocytes, or interact with signal receptors such as DR5 and TGF-βR1. This could influence epithelial cells or the surrounding matrix and the immune microenvironment, altering their signaling pathways to promote metastasis (30).

Acute phase reactant proteins (APPs), such as positive APPs [including α1-antitrypsin (AAT), α1-antichymotrypsin, haptoglobin, ceruloplasmin, C3, and C-reactive protein (CRP)] and negative APPs (comprising albumin, prealbumin, and transferrin) serve as inflammatory biomarkers predicting cancer patient outcomes (32). Serum protein electrophoresis profiles, indicative of APR by changes in serum protein fraction bands, are characterized by α1-globulin for AAT and α1-antichymotrypsin, α2-globulin for haptoglobin and ceruloplasmin, β1-globulin for C3, transferrin, and hemopexin, and γ-globulin for CRP (32, 33). The increase of positive APPs and the decrease of negative APPs illustrated the activation of APR, which plays a role in the progression of cancer (34). Interestingly, we find the α1-globulin values is beyond the normal range, while α1-globulin SPF band is mainly composed of α1-antitrypsin (AAT) and other proteins, including high-density lipoprotein, α1-antichymotrypsin, and α-fetoprotein (32). Serum AAT have immunosuppressive properties by suppressing lymphocyte proliferation, decreasing natural killing (NK) cells and inhibiting antibody-dependent cell-mediated cytotoxicity (35, 36). The immune inflammation index also showed significant changes in DPGC, as peripheral blood lymphocytes decreased significantly, indicating the presence of immune disorder in DPGC.

The results of clinical studies have confirmed the role of combination of immunotherapy and chemotherapy in the first-line treatment of advance gastric cancer (37). However, to date, no literature has reported the efficacy of immunotherapy combined with chemotherapy in patients with AFPGC. We found that the combination of immunotherapy with chemotherapy resulted in or significant benefit compared to chemotherapy alone, and the beneficial effect was more pronounced in the DPGC subgroup. In our study cohort, the immunotherapeutic agents were inhibitors of PD-1 or PD-L1. PD-L1, present on both antigen-presenting cells and tumor cells, interacts with PD-1 to initiate immunosuppressive signaling cascades that result in T-cell dysfunction and tumor immune evasion. Consequently, inhibitors targeting the PD-1/PD-L1 axis have the potential to bolster the immune system’s response to malignancies by disrupting this interaction (38). We speculate that the significant benefit derived from immunotherapy in DPGC patients may be associated with the restoration of immune homeostasis by PD-1/PD-L1 inhibitors, although the specific mechanisms involved remain unknown.

Our study is subject to several limitations that need to be acknowledged. First, the retrospective nature of our study led to incomplete data in some areas, which introduced limitations and unfortunately necessitated the exclusion of certain potentially informative indicators. Prospective studies are warranted to explore these indicators more thoroughly. Also, despite being the largest single-center study of its kind for AFPGC, the sample size for DPGC patients remains small, hindering a robust analysis of the impact of current treatment strategies on prognosis. A multicenter collaborative effort is needed to fully explore the treatment response of DPGC patients and to develop new therapeutic options.

Above all, DPGC is an extremely malignant gastric cancer with poor survival outcome. We suggest that the poor prognosis can be ascribed to tumor local characteristics together with systemic characteristics, including systemic inflammation, cancer-associated thrombosis and malnutrition. Elevated serum CEA in AFPGC is mainly associated with liver metastasis and elevated neutrophils. DPGC patients could benefit from immunotherapy in combination with chemotherapy.

## Data Availability

The original contributions presented in the study are included in the article/supplementary material. Further inquiries can be directed to the corresponding authors.
